# Conservation status of native plant hybrids in the British Virgin Islands

**DOI:** 10.3897/BDJ.9.e62809

**Published:** 2021-03-15

**Authors:** Sara Bárrios, Martin A Hamilton

**Affiliations:** 1 Royal Botanic Gardens, Kew, London, United Kingdom Royal Botanic Gardens, Kew London United Kingdom

**Keywords:** Caribbean flora, hybrid plant species, conservation status, endemism

## Abstract

**Background:**

Hybridization is an evolutionary event present in the natural world. Several studies suggest that natural hybridization is an important process in plant evolution, creating new genetic combinations which can play a vital role in speciation (Soltis and Soltis 2009, Soltis 2013, Neri et al. 2017, Taylor and Larson 2019). Therefore, it is important to understand and protect naturally occurring hybrids, conserving their ecological novelties and new traits, such as the ability to explore new niches, different from those of the parental species (Soltis 2013, Supple and Shapiro 2018).

The British Virgin Islands (BVI) is a UK Overseas Territory situated in the Caribbean biodiversity hotspot (Myers et al. 2000). To date, three natural hybrids are known to occur within this territory: Tillandsia × lineatispica Mez, Anthurium × selloanum K.Koch and *Coccoloba krugii* × *C. uvifera* R.A.Howard (Howard 1957, Acevedo-Rodriguez and Strong 2005, Acevedo-Rodriguez and Strong 2012).

Tillandsia × lineatispica is endemic to the Puerto Rican Bank, occurring in Puerto Rico, the US Virgin Islands (USVI) and the British Virgin Islands with an extent of occurrence estimated to be 3,390 km^2^ and a limited number of locations. The suitable habitat for this hybrid is declining mainly due to the negative impacts of feral ungulates, development for tourism and residential infrastructure and the impact of human-induced wildfires. In addition, it is suspected that the global population does not exceed 10,000 individuals with the largest subpopulation on Beef Island in the BVI thought to have no more than 1,000 mature individuals. This hybrid is therefore evaluated as Vulnerable, based on IUCN Red List Criteria, B1a(iii)+2b(iii) + C2a(i).

Anthurium × selloanum is an endemic hybrid to BVI and USVI with a very restricted extent of occurrence which was estimated to range between 103 km^2^ and 207 km^2^ and an area of occupancy which was estimated to range between 56 km^2^ and 188 km^2^ and a limited number of locations. The suitable habitat of this species is declining mainly due to the negative impacts of feral ungulates, development for tourism and residential infrastructure and the negative impact of recreation activities in protected areas. This species is therefore evaluated as Endangered, based on IUCN Red List Criteria B1a+ b(iii) + B2a+b(iii).

*Coccoloba krugii* × *C. uvifera* is native to the BVI, USVI, Puerto Rico, Dominican Republic, Haiti and Anguilla. It is estimated to have an extent of occurrence of 89,412 km^2^. This value exceeds the threshold for any threatened category. Despite an observed continuing decline of suitable habitat for this species, which is being degraded mainly through ongoing development pressures, this species occurs in more than 10 locations. It is therefore assessed as Least Concern (LC).

**New information:**

In this paper, we discuss the conservation status of all the known, naturally occurring, native hybrids in the the British Virgin Islands and we provide distribution data, including new records, from across these hybrid species ranges. Although conservation assessments of hybrids are out of the scope of the published IUCN Red List of Threatened Species (IUCN Standards and Petitions Committee 2019), we use the IUCN Red List Criteria and Categories (version 3.1) to establish an equivalent conservation status of these hybrids and discuss conservation action due to the potential evolutionary importance of these naturally occurring hybrids. These assessments provide the necessary baseline information for prioritising species conservation and making informed management decisions, such as establishing the BVI's Tropical Important Plant Areas (TIPAS) network (Sanchez et al. 2019).

## Introduction

To map the distribution range of these hybrids, literature records and previously existing records in several herbaria (K, NY, US, UPR, SJ, MO) have been digitised and complemented with records made during field surveys, which took place beween 2014 and 2020 in the British Virgin Islands (BVI), the US Virgin Islands (USVI) and Puerto Rico. Field surveys were also used to record observed threats and evaluate population sizes.

## Species Conservation Profiles

### Tillandsia × lineatispica

#### Species information

Scientific name: Tillandsia × lineatispica

Species authority: Mez

Kingdom: Plantae

Phylum: Tracheophyta

Class: Liliopsida

Order: Polaes

Family: Bromeliaceae

Taxonomic notes: This species is considered to be a sterile hybrid between *T. utriculata* and *T. fasciculata* (Acevedo-Rodriguez and Strong 2005, Acevedo-Rodriguez and Strong 2012).

Region for assessment: Global

#### Editor & Reviewers

##### Reviewers

Reviewers: 

##### Editor

Editor: 

#### Reviewers

Reviewers: 

#### Editor

Editor: 

#### Geographic range

Biogeographic realm: Neotropical

Countries: Virgin Islands, BritishVirgin Islands, U.S.Puerto Rico

Map of records (Google Earth): 

Basis of EOO and AOO: Observed

Basis (narrative): The species extent of occurrence (EOO), based on known collections and literature records is estimated to be 3,390 km^2^ and a minimum area of occupancy (AOO), based on known collections, to be 52 km^2^ using a 2 x 2 km cell size. EOO and AOO were calculated using GeoCAT (Bachman et al. 2011).

Min Elevation/Depth (m): 10

Max Elevation/Depth (m): 400

**Figure 1.**
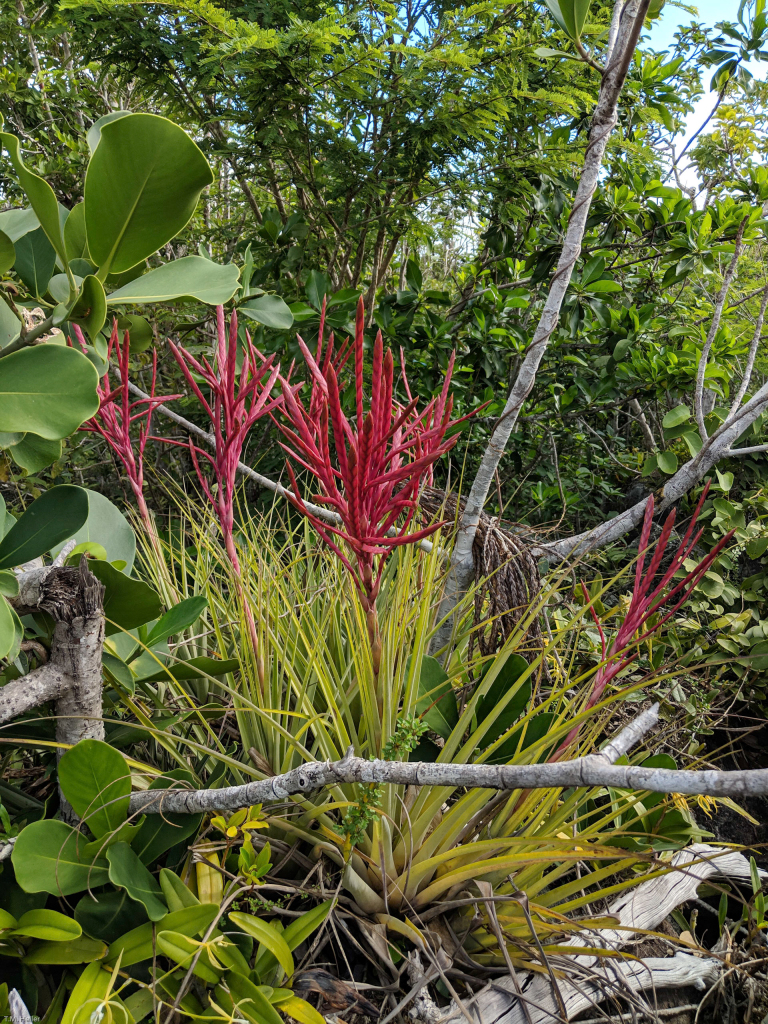
Tillandsia × lineatispica Mez, with a distinctive, coral-coloured sterile inflorescence.

Range description: Tillandsia × lineatispica (Fig. 1) is a hybrid restricted to the British Virgin Islands (BVI), the US Virgin Islands (USVI) and to the Commonwealth of Puerto Rico (Acevedo-Rodriguez and Strong 2005, Axelrod 2011, Hamilton et al. 2016). In the BVI, this species is known from a single locality on the island of Tortola, Mount Alma on Beef Island and Gorda Peak and Little Fort National Parks on the island of Virgin Gorda (Hamilton et al. 2016). In the USVI, collections show that this species is found exclusively on the western part of the island of St John. In the Commonwealth of Puerto Rico, this hybrid occurs on the island of Puerto Rico in Guánica State Forest and is also known from the islands of Culebra and Vieques (Acevedo-Rodriguez and Strong 2005, Axelrod 2011). This species is not considered to be severely fragmented (Suppl. material 1).

#### New occurrences

#### Extent of occurrence

EOO (km2): 3390 km^2^

Trend: Stable

Causes ceased?: Unknown

Causes understood?: Unknown

Causes reversible?: Unknown

Extreme fluctuations?: No

#### Area of occupancy

Trend: Unknown

Causes ceased?: Unknown

Causes understood?: Unknown

Causes reversible?: Unknown

Extreme fluctuations?: No

AOO (km2): 52 km^2^

#### Locations

Number of locations: 7

Justification for number of locations: The number of locations was calculated to be seven considering the main threats to the species, namely fire, development and feral ungulates, which can vary by island.

Trend: Stable

Extreme fluctuations?: No

#### Population

Number of individuals: Unknown

Trend: Unknown

Causes ceased?: Yes

Causes understood?: Yes

Causes reversible?: Yes

Extreme fluctuations?: No

Population Information (Narrative): This hybrid is considered rare across its entire range (Acevedo-Rodriguez 1996, Acevedo-Rodriguez and Strong 2005). Precise numbers for each subpopulation are unknown. Based on existing herbarium collections and observation records, the largest subpopulation occurs on Mount Alma, Beef Island in the BVI. However, this hybrid is still considered rare at this location with considerably less than 1,000 individuals observed (Hamilton et al. 2016).

#### Subpopulations

Trend: Decline (observed)

Extreme fluctuations?: No

Severe fragmentation?: No

#### Habitat

System: Terrestrial

Habitat specialist: No

Habitat (narrative): This hybrid is a terrestrial or lithophytic acaulescent herb with leaves growing in a rosette between 60 to 95 cm long. The apparently sterile, twisted inflorescence is coral-coloured. It prefers dry forest habitat, growing mainly on rocky outcrops from sea level to higher elevations (Acevedo-Rodriguez and Strong 2005), predominantly in coastal areas less than 400 m above sea level.

Trend in extent, area or quality?: Decline (observed)

Justification for trend: The species suitable habitat is decreasing due to free ranging feral ungulates, which affect soil quality, the reproductive success of the parent species and the establishment of hybrid plants and the increasing pressure from development of residential and tourism infrastructure.

Figure(s) or Photo(s): Fig. 1

##### Habitat

Habitat importance: Major Importance

Habitats: 1.5. Forest - Subtropical/Tropical Dry3.5. Shrubland - Subtropical/Tropical Dry

#### Habitat

Habitat importance: Major Importance

Habitats: 1.5. Forest - Subtropical/Tropical Dry3.5. Shrubland - Subtropical/Tropical Dry

#### Ecology

Generation length (yr): 0

Dependency of single sp?: Unknown

Ecology and traits (narrative): Generation length is not applicable for sterile hybrids.

#### Threats

Justification for threats: Feral livestock, especially goats (*Capra hircus*), across all islands in the BVI and feral deer (*Odocoileus virginianus*) on St John are grazing on forest species and degrading and altering soil quality, contributing to a decline in suitable habitat. Throughout the hybrid species range, its suitable habitat is highly fragmented due to the increase in developed areas, mainly for housing, tourism and recreational infrastructure. Road improvements and land clearance, which contribute to habitat fragmentation, have also been observed. Within Guánica State Forest on the island of Puerto Rico, this hybrid might be affected by human induced forest fires which are frequent at this location. Climate change might already be impacting the species through prolonged periods of drought.

##### Threats

Threat type: Ongoing

Threats: 1.3. Residential & commercial development - Tourism & recreation areas4.2. Transportation & service corridors - Utility & service lines7.1. Natural system modifications - Fire & fire suppression8.1.2. Invasive and other problematic species, genes & diseases - Invasive non-native/alien species/diseases - Named species11.1. Climate change & severe weather - Habitat shifting & alteration

#### Threats

Threat type: Ongoing

Threats: 1.3. Residential & commercial development - Tourism & recreation areas4.2. Transportation & service corridors - Utility & service lines7.1. Natural system modifications - Fire & fire suppression8.1.2. Invasive and other problematic species, genes & diseases - Invasive non-native/alien species/diseases - Named species11.1. Climate change & severe weather - Habitat shifting & alteration

#### Conservation

Justification for conservation actions: This hybrid is known to occur in several existing protected areas within its natural range with almost 50% of the known individuals under protection. In the BVI, this hybrid is recorded in Gorda Peak National Park and Little Fort National Park on the island of Virgin Gorda. In the USVI, it occurs within the Virgin Islands National Park on the island of St John. In the Commonwealth of Puerto Rico, this hybrid occurs inside the boundaries of Guánica State Forest on the island of Puerto Rico and in Vieques National Wildlife Refuge on the island of Vieques. In the BVI, this species occurs within the designated Beef Island and the Channel BVI TIPA (Sanchez et al. 2019). There are no known *ex situ* collections for this hybrid. Conservation actions should focus on habitat management in areas suitable for the hybrid and its parent species.

##### Conservation actions

Conservation action type: Needed

Conservation actions: 1.2. Land/water protection - Resource & habitat protection2.2. Land/water management - Invasive/problematic species control3.4. Species management - Ex-situ conservation

##### Conservation actions

Conservation action type: In Place

Conservation actions: 1.1. Land/water protection - Site/area protection

#### Conservation actions

Conservation action type: Needed

Conservation actions: 1.2. Land/water protection - Resource & habitat protection2.2. Land/water management - Invasive/problematic species control3.4. Species management - Ex-situ conservation

#### Conservation actions

Conservation action type: In Place

Conservation actions: 1.1. Land/water protection - Site/area protection

#### Other

Justification for use and trade: There are no known uses for this hybrid.

##### Use and trade

Use type: National

##### Ecosystem services

Ecosystem service type: Very important

##### Research needed

Research needed: 1.2. Research - Population size, distribution & trends1.3. Research - Life history & ecology2.2. Conservation Planning - Area-based Management Plan3.1. Monitoring - Population trends

Justification for research needed: Detailed surveys across the species range should be undertaken to document precise numbers of mature individuals per subpopulation. Monitoring is required to record phenology of wild populations and *ex-situ* collections should be established to enable detailed studies of the species reproductive structures. Further research into the hybrid and parent species life histories is needed.

#### Use and trade

Use type: National

#### Ecosystem services

Ecosystem service type: Very important

#### Research needed

Research needed: 1.2. Research - Population size, distribution & trends1.3. Research - Life history & ecology2.2. Conservation Planning - Area-based Management Plan3.1. Monitoring - Population trends

Justification for research needed: Detailed surveys across the species range should be undertaken to document precise numbers of mature individuals per subpopulation. Monitoring is required to record phenology of wild populations and *ex-situ* collections should be established to enable detailed studies of the species reproductive structures. Further research into the hybrid and parent species life histories is needed.

#### Viability analysis

### Anthurium × selloanum

#### Species information

Scientific name: Anthurium × selloanum

Species authority: K.Koch

Kingdom: Plantae

Phylum: Tracheophyta

Class: Liliopsida

Order: Polaes

Family: Bromeliaceae

Taxonomic notes: According to article 60.8(c) of the International Code of Nomenclature for algae, fungi and plants (Turland et al. 2018), this species name must be corrected from Anthurium × selloum to Anthurium × selloanum, since it is named after Mr. Sello (Rafaël Govaerts, pers. comm. 2019). This hybrid is the result of a crossing between *A. cordatum* and *A. crenatum*. It has never been described, collected or observed in fruit (Acevedo-Rodriguez 1996).

Region for assessment: Global

#### Editor & Reviewers

##### Reviewers

Reviewers: 

##### Editor

Editor: 

#### Reviewers

Reviewers: 

#### Editor

Editor: 

#### Geographic range

Biogeographic realm: Neotropical

Countries: Virgin Islands, BritishVirgin Islands, U.S.

Map of records (Google Earth): 

Basis of EOO and AOO: Observed

Basis (narrative): Known herbarium collections and recent observations were considered to calculate minimum values of extent of occurrence (EOO) and area of occupancy (AOO), while maximum values of EOO and AOO were calculated considering the whole island of Tortola in the BVI and the whole island of St John in the USVI, since a detailed distribution for this hybrid is unknown and it is suspected to be widespread across these islands. The extent of occurrence (EOO) was estimated to range between 103 km^2^ and 207 km^2^. The area of occupancy (AOO) was estimated to range between 56 km^2^ and 188 km^2^. Both calculations for EOO and AOO are based on a 2 x 2 km cell size and were calculated with GeoCAT (Bachman et al. 2011).

Min Elevation/Depth (m): 15

Max Elevation/Depth (m): 500

**Figure 2.**
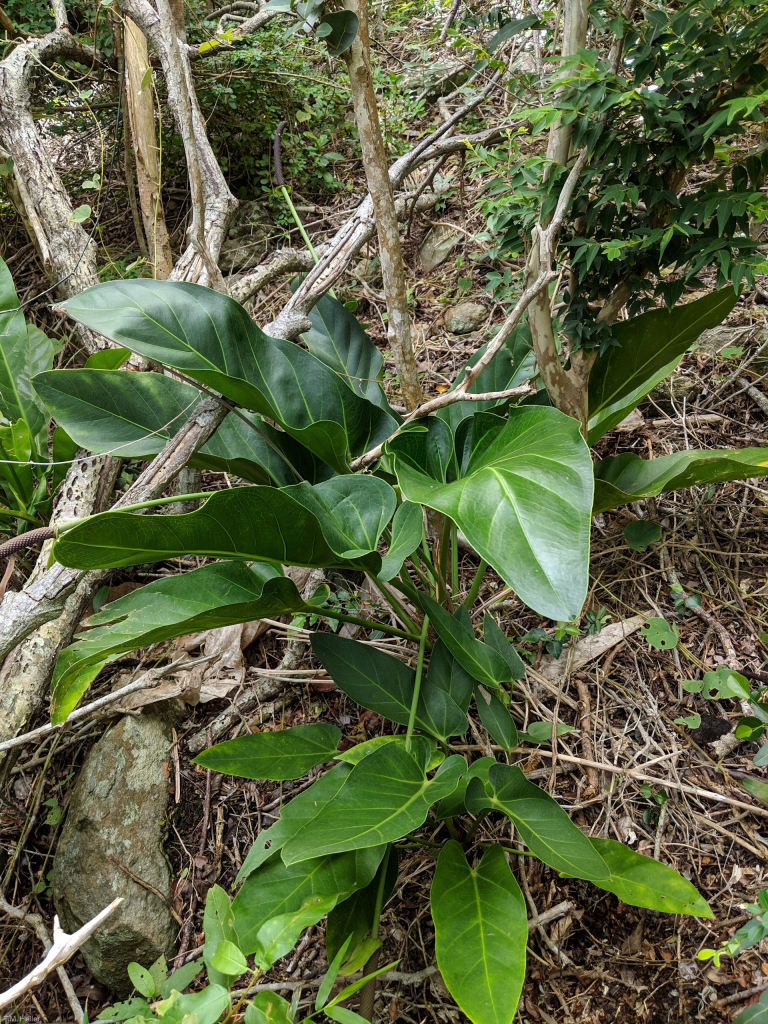
Anthurium × selloanum K.Koch.

Range description: Anthurium × selloanum (Fig. 2) is an endemic hybrid to the BVI and the USVI. In the BVI, this hybrid is only found on the island of Tortola, while in the USVI, it occurs exclusively on the island of St John (Acevedo-Rodriguez 1996, Suppl. material 2).

#### New occurrences

#### Extent of occurrence

EOO (km2): 103-207 km^2^

Trend: Unknown

Causes ceased?: Unknown

Causes understood?: Unknown

Causes reversible?: Unknown

Extreme fluctuations?: No

#### Area of occupancy

Trend: Unknown

Causes ceased?: Unknown

Causes understood?: Unknown

Causes reversible?: Unknown

Extreme fluctuations?: No

AOO (km2): 56-188 km^2^

#### Locations

Number of locations: 5

Justification for number of locations: The number of locations was calculated to be five, considering threats posed by feral ungulates and development, which can vary depending on whether this hybrid is found within or outside protected areas on each island.

Trend: Unknown

Extreme fluctuations?: No

#### Population

Number of individuals: Unknown

Trend: Unknown

Causes ceased?: Unknown

Causes understood?: Unknown

Causes reversible?: Unknown

Extreme fluctuations?: No

Population Information (Narrative): This hybrid is considered common across its range (Acevedo-Rodriguez 1996). Precise numbers for each subpopulation are unknown and further surveys are needed to confirm the total number of individuals.

#### Subpopulations

Trend: Decline (observed)

Extreme fluctuations?: No

Severe fragmentation?: No

#### Habitat

System: Terrestrial

Habitat specialist: No

Habitat (narrative): This hybrid is a terrestrial or epiphytic herb, 0.5-1 m tall and with numerous adventitious roots. It prefers the moist to dry forest habitat, growing in shady places from sea level to almost 500 metres above sea level (Acevedo-Rodriguez 1996).

Trend in extent, area or quality?: Decline (observed)

Justification for trend: The suitable habitat is decreasing due to free ranging feral ungulates, which feed directly on this hybrid and affect soil quality and the increasing pressure from development of residential and tourism infrastructure.

##### Habitat

Habitat importance: Major Importance

Habitats: 1.5. Forest - Subtropical/Tropical Dry1.6. Forest - Subtropical/Tropical Moist Lowland

#### Habitat

Habitat importance: Major Importance

Habitats: 1.5. Forest - Subtropical/Tropical Dry1.6. Forest - Subtropical/Tropical Moist Lowland

#### Ecology

Generation length (yr): 0

Dependency of single sp?: Unknown

Ecology and traits (narrative): Generation length is not applicable for sterile hybrids.

#### Threats

Justification for threats: Feral livestock, especially goats (*Capra hircus*) on Tortola and feral deer (*Odocoileus virginianus*) on St John, are grazing on forest plants and degrading and altering soil quality, contributing to a decline in suitable habitat. Development for housing and tourism are further fragmenting suitable habitat throughout the distribution range of the hybrid. Road improvements and installations and land clearance for new development projects, which contribute to habitat fragmentation, have also been observed. Climate change might already be impacting this hybrid through prolonged periods of drought.

##### Threats

Threat type: Ongoing

Threats: 1.1. Residential & commercial development - Housing & urban areas1.3. Residential & commercial development - Tourism & recreation areas4.2. Transportation & service corridors - Utility & service lines6.1. Human intrusions & disturbance - Recreational activities8.1.2. Invasive and other problematic species, genes & diseases - Invasive non-native/alien species/diseases - Named species11.2. Climate change & severe weather - Droughts

#### Threats

Threat type: Ongoing

Threats: 1.1. Residential & commercial development - Housing & urban areas1.3. Residential & commercial development - Tourism & recreation areas4.2. Transportation & service corridors - Utility & service lines6.1. Human intrusions & disturbance - Recreational activities8.1.2. Invasive and other problematic species, genes & diseases - Invasive non-native/alien species/diseases - Named species11.2. Climate change & severe weather - Droughts

#### Conservation

Justification for conservation actions: This hybrid is known to occur in existing protected areas within its natural range with almost 35% of the known individuals under protection. In the BVI, this hybrid is recorded in Sage Mountain National Park and Shark Bay National Park on the island of Tortola. In the USVI, it occurs within the Virgin Islands National Park on the island of St John. In the BVI, this species occurs within the designated Tortola North Shore BVI TIPA (Sanchez et al. 2019). Conservation actions should mainly focus on control of invasive mammals, monitoring the suitable habitat of this hybrid and its parent species and further research is required into this hybrid species traits and ecology to understand its role in the wider ecosystem.

##### Conservation actions

Conservation action type: Needed

Conservation actions: 1.1. Land/water protection - Site/area protection1.2. Land/water protection - Resource & habitat protection2.2. Land/water management - Invasive/problematic species control3.4. Species management - Ex-situ conservation4.3. Education & awareness - Awareness & communications

##### Conservation actions

Conservation action type: In Place

Conservation actions: 1.1. Land/water protection - Site/area protection

#### Conservation actions

Conservation action type: Needed

Conservation actions: 1.1. Land/water protection - Site/area protection1.2. Land/water protection - Resource & habitat protection2.2. Land/water management - Invasive/problematic species control3.4. Species management - Ex-situ conservation4.3. Education & awareness - Awareness & communications

#### Conservation actions

Conservation action type: In Place

Conservation actions: 1.1. Land/water protection - Site/area protection

#### Other

Justification for use and trade: There are no known uses for this hybrid.

##### Use and trade

Use type: National

##### Ecosystem services

Ecosystem service type: Very important

##### Research needed

Research needed: 1.2. Research - Population size, distribution & trends1.3. Research - Life history & ecology2.1. Conservation Planning - Species Action/Recovery Plan

Justification for research needed: Detailed surveys should take place to document precise numbers of mature individuals per subpopulation. Research into the species life history is required to confirm if it is fertile. The establishment of *ex-situ* collections should be prioritised to enable detailed studies of the species phenology.

#### Use and trade

Use type: National

#### Ecosystem services

Ecosystem service type: Very important

#### Research needed

Research needed: 1.2. Research - Population size, distribution & trends1.3. Research - Life history & ecology2.1. Conservation Planning - Species Action/Recovery Plan

Justification for research needed: Detailed surveys should take place to document precise numbers of mature individuals per subpopulation. Research into the species life history is required to confirm if it is fertile. The establishment of *ex-situ* collections should be prioritised to enable detailed studies of the species phenology.

#### Viability analysis

### Coccoloba krugii × C. uvifera

#### Species information

Scientific name: Coccoloba krugii × C. uvifera

Species authority: R.A.Howard

Kingdom: Plantae

Phylum: Tracheophyta

Class: Magnoliopsida

Order: Ploygonales

Family: Polygonaceae

Taxonomic notes: This hybrid swarm is a result of the crossing of *C. krugii* and *C. uvifera* (Howard 1957). The hybrid name is not formally recognised by the International Plant Names Index (IPNI). Further taxonomic work, which includes samples from across its range, needs to be included in phylogenomic studies to resolve taxonomic relationships.

Region for assessment: Global

#### Editor & Reviewers

##### Reviewers

Reviewers: 

##### Editor

Editor: 

#### Reviewers

Reviewers: 

#### Editor

Editor: 

#### Geographic range

Biogeographic realm: Neotropical

Countries: Virgin Islands, BritishVirgin Islands, U.S.HaitiDominican RepublicAnguillaPuerto Rico

Map of records (Google Earth): 

Basis of EOO and AOO: Observed

Basis (narrative): The extent of occurrence (EOO) was estimated to be 89,412 km^2^. A minimum area of occupancy (AOO), based on known herbarium collections and observation records, was calculated to be 68 km^2^, considering a 2 x 2 km cell size, calculated with GeoCAT (Bachman et al. 2011).

Min Elevation/Depth (m): 10

Max Elevation/Depth (m): 400

**Figure 3.**
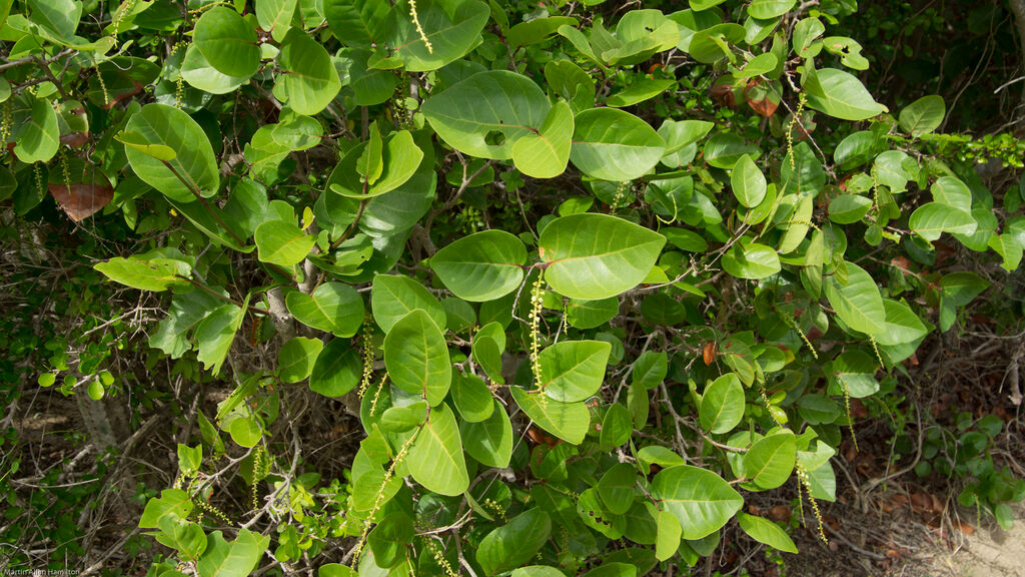
Shrub of *Coccoloba krugii* × *C. uvifera* R.A.Howard.

Range description: *Coccoloba krugii* × *C. uvifera* (Fig. 3) is a hybrid plant that naturally occurs in the BVI, USVI, Puerto Rico, Dominican Republic and Haiti in the Greater Antilles and the island of Anguilla in the Lesser Antilles (*Howard 1957, Howard 1958, Howard and Kellogg 1987, Axelrod 2011, Acevedo-Rodriguez and Strong 2012*). In the BVI, this hybrid is found on Virgin Gorda and Scrub Island (Hamilton et al. 2019), while in the USVI, it occurs on the islands of St John and St. Croix (Acevedo-Rodriguez and Strong 2012). In the Commonwealth of Puerto Rico, this hybrid is known from Culebra, Vieques, Mona and Desecheo islands and from the Fajardo area on the island of Puerto Rico (Axelrod 2011). In the Dominican Republic and Haiti on the island of Hispaniola, herbarium collections suggest that this hybrid occurs along the Northwest coast, in the Cayos Siete Hermanos refuge and the Nord-Ouest Province. The precise location of the herbarium collections and observations from Anguilla, cited by Howard and Kellogg (1987), is not known (Suppl. material 3).

#### New occurrences

#### Extent of occurrence

EOO (km2): 89412

Trend: Unknown

Causes ceased?: Yes

Causes understood?: Yes

Causes reversible?: Yes

Extreme fluctuations?: No

#### Area of occupancy

Trend: Unknown

Causes ceased?: Yes

Causes understood?: Yes

Causes reversible?: Yes

Extreme fluctuations?: No

AOO (km2): 68

#### Locations

Number of locations: 16

Justification for number of locations: A minimum number of locations was calculated to be 16, considering threats posed by development, which can vary depending on whether this hybrid is found within or outside protected areas on each island. Field observations suggest that this hybrid is not abundant, but it is possible that further surveys in poorly known areas, such as Anguilla or Dominican Republic, will increase the number of known individuals, as well as the AOO and number of locations.

Trend: Unknown

Extreme fluctuations?: No

#### Population

Number of individuals: Unknown

Trend: Unknown

Causes ceased?: Yes

Causes understood?: Yes

Causes reversible?: Yes

Extreme fluctuations?: No

Population Information (Narrative): Precise numbers for each subpopulation are unknown and further surveys are needed to estimate the total number of individuals. The suitable habitat for this hybrid is decreasing due to the increasing pressure from development of residential and tourism infrastructure.

#### Subpopulations

Trend: Decline (observed)

Extreme fluctuations?: No

Severe fragmentation?: No

#### Habitat

System: Terrestrial

Habitat specialist: No

Habitat (narrative): This hybrid is a shrub or a small tree which does not produce viable fruits. It grows in coastal areas of dry forest. Herbarium collections suggest that this hybrid grows from sea level to almost 500 m above sea level.

Trend in extent, area or quality?: Decline (observed)

##### Habitat

Habitat importance: Major Importance

Habitats: 1.5. Forest - Subtropical/Tropical Dry3.5. Shrubland - Subtropical/Tropical Dry

#### Habitat

Habitat importance: Major Importance

Habitats: 1.5. Forest - Subtropical/Tropical Dry3.5. Shrubland - Subtropical/Tropical Dry

#### Ecology

Generation length (yr): 0

Dependency of single sp?: Unknown

Ecology and traits (narrative): Generation length is not applicable for sterile hybrids.

#### Threats

Justification for threats: Across its range, development for housing and tourism and impacts from recreational activities are fragmenting the species suitable habitat. Road improvements and land clearance, which contribute to habitat fragmentation, have also been observed. Recreational activities, trail cutting and use of all-terrain vehicles, have also been observed in areas where this species occurs, causing negative impacts on the native vegetation. Climate change might already be impacting this hybrid through prolonged periods of drought, sea level rise and increased intensity of tropical storms.

##### Threats

Threat type: Ongoing

Threats: 1.1. Residential & commercial development - Housing & urban areas1.3. Residential & commercial development - Tourism & recreation areas6.1. Human intrusions & disturbance - Recreational activities11.2. Climate change & severe weather - Droughts

#### Threats

Threat type: Ongoing

Threats: 1.1. Residential & commercial development - Housing & urban areas1.3. Residential & commercial development - Tourism & recreation areas6.1. Human intrusions & disturbance - Recreational activities11.2. Climate change & severe weather - Droughts

#### Conservation

Justification for conservation actions: This hybrid is known to occur in existing protected areas within its natural range, with only around 15% of the known individuals under protection. In the BVI, this hybrid is recorded in Gorda Peak National Park on the island of Virgin Gorda. In the USVI, it occurs within the Virgin Islands National Park on the island of St John and on Buck Island within the Buck Island Reef National Monument, near St Croix. In the Commonwealth of Puerto Rico, this hybrid occurs within protected areas on Mona, Desecheo, Culebra and Vieques islands. In the Dominican Republic, it occurs within a wildlife refuge, Cayos Siete Hermanos. Conservation actions should focus on protection of the suitable habitat of this hybrid and its parent species.

##### Conservation actions

Conservation action type: Needed

Conservation actions: 1.2. Land/water protection - Resource & habitat protection4.3. Education & awareness - Awareness & communications

##### Conservation actions

Conservation action type: In Place

Conservation actions: 1.1. Land/water protection - Site/area protection

#### Conservation actions

Conservation action type: Needed

Conservation actions: 1.2. Land/water protection - Resource & habitat protection4.3. Education & awareness - Awareness & communications

#### Conservation actions

Conservation action type: In Place

Conservation actions: 1.1. Land/water protection - Site/area protection

#### Other

Justification for use and trade: There are no known uses for this hybrid.

##### Use and trade

Use type: International

##### Ecosystem services

Ecosystem service type: Very important

##### Research needed

Research needed: 1.1. Research - Taxonomy1.2. Research - Population size, distribution & trends1.3. Research - Life history & ecology1.5. Research - Threats2.2. Conservation Planning - Area-based Management Plan3.1. Monitoring - Population trends3.4. Monitoring - Habitat trends

Justification for research needed: Conservation research should focus on detailed surveys to document precise numbers per subpopulation. Research into the life history of the hybrid is required to confirm if it is reproductive. Phylogenomic studies and taxonomic revision, which includes material from across the known range of the parent species and the hybrid swarm is needed.

#### Use and trade

Use type: International

#### Ecosystem services

Ecosystem service type: Very important

#### Research needed

Research needed: 1.1. Research - Taxonomy1.2. Research - Population size, distribution & trends1.3. Research - Life history & ecology1.5. Research - Threats2.2. Conservation Planning - Area-based Management Plan3.1. Monitoring - Population trends3.4. Monitoring - Habitat trends

Justification for research needed: Conservation research should focus on detailed surveys to document precise numbers per subpopulation. Research into the life history of the hybrid is required to confirm if it is reproductive. Phylogenomic studies and taxonomic revision, which includes material from across the known range of the parent species and the hybrid swarm is needed.

#### Viability analysis

## Conclusion

These three plant hybrids are exposed to the same threats as other plant species in the region (Heller 2019). As such, they are losing suitable habitat and their remaining habitat is being fragmented and degraded, mainly due to the encroachment of developement and the action of feral ungulates that roam free across these plant hybrids native range. Conserving these plant hybrids and their parent species and securing their suitable habitat is essential to safeguard these plants across this Caribbean biodiversity hotspot.

Conclusion

## Supplementary Material

9C2120D2-19E1-512F-961A-8C75E2986699 10.3897/BDJ.9.e62809.suppl1 Supplementary material 1 Google Map showing existing records of Tillandsia × lineatispica Mez Data type Occurrences Brief description Known records of Tillandsia × lineatispica Mez File: oo_359918.kml https://binary.pensoft.net/file/359918 Barrios, S. & Hamilton, M.A.

EB405A25-2A8E-5644-884B-57E1FE3F0DCA 10.3897/BDJ.9.e62809.suppl2 Supplementary material 2 Google map showing existing records for Anthurium × selloanum K.Koch Data type Occurrences Brief description Known records of Anthurium × selloanum K.Koch File: oo_359919.kml https://binary.pensoft.net/file/359919 Barrios, S. & Hamilton, M.A.

08B8847A-6584-5B8F-B70E-865508A0C1C2 10.3897/BDJ.9.e62809.suppl3 Supplementary material 3 Google map showing existing records for Coccoloba krugii × uvifera R.A. Howard Data type Occurences Brief description Known occurrences for Coccoloba krugii × uvifera R.A. Howard. File: oo_359927.kml https://binary.pensoft.net/file/359927 Barrios, S. & Hamilton. M.A.
